# A technique for non-intrusive greedy piecewise-rational model reduction of frequency response problems over wide frequency bands

**DOI:** 10.1186/s13362-021-00117-4

**Published:** 2022-01-03

**Authors:** Davide Pradovera, Fabio Nobile

**Affiliations:** 1 CSQI, EPFL, MA B2 435 (Bâtiment MA), Station 8, 1015 Lausanne, Switzerland; 2 CSQI, EPFL, MA B2 434 (Bâtiment MA), Station 8, 1015 Lausanne, Switzerland

**Keywords:** 35B30, 35P15, 41A20, 93C80, Model order reduction, Rational interpolation, Greedy algorithm

## Abstract

In the field of model order reduction for frequency response problems, the minimal rational interpolation (MRI) method has been shown to be quite effective. However, in some cases, numerical instabilities may arise when applying MRI to build a surrogate model over a large frequency range, spanning several orders of magnitude. We propose a strategy to overcome these instabilities, replacing an unstable global MRI surrogate with a union of stable local rational models. The partitioning of the frequency range into local frequency sub-ranges is performed automatically and adaptively, and is complemented by a (greedy) adaptive selection of the sampled frequencies over each sub-range. We verify the effectiveness of our proposed method with two numerical examples.

## Introduction

The numerical simulation of frequency-domain dynamical systems is crucial in many engineering applications, spanning from the analysis of the natural modes of elastic structures to the frequency response of electrical circuits. Under linearity assumptions, we can cast such problems in the general form

1 $$\begin{aligned} \textstyle\begin{cases} A(z)x(z)=B(z), \\ y(z)=C(z)x(z)+D(z), \end{cases}\displaystyle \end{aligned}$$

with $z\in {\mathbb{C}}$ the complex frequency. We denote by $x(z)\in {\mathbb{C}}^{n_{S}\times n_{I}}$ and $y(z)\in {\mathbb{C}}^{n_{O}\times n_{I}}$ the system state and output, respectively, whereas $A(z)\in {\mathbb{C}}^{n_{S}\times n_{S}}$, $B(z)\in {\mathbb{C}}^{n_{S}\times n_{I}}$, $C(z)\in {\mathbb{C}}^{n_{O}\times n_{S}}$, and $D(z)\in {\mathbb{C}}^{n_{O}\times n_{I}}$ are *z*-dependent matrices.

In the simplest case, e.g., when the time-domain version of the system is linear and time-invariant, the matrix *A* depends at most polynomially on the frequency, with the most common case being the degree-1 one:

2 $$\begin{aligned} A(z)=A_{0}+zE. \end{aligned}$$

It is useful to note that, even if *A* is not of the form (2), it is sometimes possible to recover a degree-1 form by augmentation, i.e., by increasing the size of the system.

### The model reduction endeavor

Assume that our target is the frequency response analysis of a quantity of interest (QoI) associated with system (1) (most often, the state *x* or the linear output *y*). To this aim, we have to evaluate the QoI at many values of the frequency, located within a “frequency range of interest” $Z\subset {\mathbb{C}}$, often an interval on the imaginary axis. This entails solving system (1) many times, thus (potentially) making the overall procedure computationally expensive. Efficiency is a concern especially when the size of the system state ($n_{S}n_{I}$) is large. To alleviate the computational burden, in the field of model order reduction (MOR), it is customary to build a surrogate model for the QoI and then use such surrogate instead of the original model when performing the frequency response analysis. We refer to [2] for an overview of the most used MOR methods for frequency response problems.

Commonly, the reduced model is constructed from (few) samples of the exact QoI at some frequencies $\{z_{1},\ldots,z_{S}\}\subset Z$. The precise way in which the surrogate is built can vary: e.g., the reduced basis method (RBM) performs a Galerkin projection of (1) onto a subspace of ${\mathbb{C}}^{n_{S}}$, whereas, in the Loewner framework and with the vector fitting (VF) method, one constructs a rational approximation by fitting (possibly in a least-squares sense) the samples. In this work, we focus on the latter class of MOR approaches, commonly referred to as *non-intrusive*, since they only rely on samples of the QoI, and not on the specific structure of the original system (1). More precisely, we focus on the minimal rational interpolation (MRI) method introduced in [5]. As opposed to most other non-intrusive methods (e.g., VF), MRI exploits the samples in an optimal way (hence the “minimal” in the name), trying to take “as few samples of the QoI as possible”.

Notably, a strategy for an *adaptive* selection of the sampled frequencies is described in [7]. Such approach, which we summarize in Sect. 2.1, has the great advantage of not requiring the user to fix the number and the locations of the sampled frequencies *in advance*, but rather finding them on-the-fly. The usefulness of this feature can be easily seen by observing that number and locations of the samples determine the cost of building and evaluating the surrogate, but also its accuracy. As such, a poor choice of the samples can lead to inaccurate or over-expensive surrogates.

Several examples of successful applications of MRI can be found in [5, 7]. However, we can identify the main drawback of MRI in its difficulty in dealing with very large frequency ranges, namely, with frequencies spanning several orders of magnitude (this is a situation of practical interest, e.g., when making Bode diagrams). Indeed, in such cases, numerical instabilities may arise, hindering the construction of a stable surrogate, as we will discuss more in detail in Sect. 2.2.

The purpose of this paper is to remedy this shortcoming of MRI, by devising a strategy that allows to apply MRI even over very large frequency ranges. Our technique is based on a partitioning of *Z* into smaller parts, such that MRI can be applied in a stable fashion over each of them. Notably, the partitioning of *Z* is performed *automatically*, in a fashion similar to that described in [4], where, however, the surrogate is built by RBM. Effectively, this allows to build a surrogate model where the only user inputs are the frequency range *Z* and a required accuracy $\varepsilon >0$. Our proposed method is described in Sect. 3. As a complement to the description of our algorithm, and as a way to validate it, we perform some numerical experiments in Sect. 4.

## The greedy minimal rational interpolation method

We start by recalling the MRI method, in its standard non-adaptive form, i.e., when the (distinct) sampled frequencies $\{z_{1},\ldots,z_{S}\}\subset Z$ are fixed in advance by the user. We restrict our discussion to the approximation of the state *x*, since MRI is most commonly used to construct a surrogate of *x* rather than of the output *y* (or of other QoIs). The reason behind this is that building a “good” surrogate via MRI is actually easier if the approximated quantity is high-dimensional. We refer to [5] for a discussion on this in the scope of a theoretical analysis of the properties of MRI. That being said, note that, thanks to the linearity of (1), an approximation *ỹ* for the output *y* can be readily and inexpensively obtained from the MRI approximation *x̃* of the state, by simply applying $C(z)$ from the left and adding $D(z)$.

Let $\{\psi _{0},\ldots,\psi _{S-1}\}\subset \mathbb{P}^{S-1}({ \mathbb{C}})$ be a hierarchical polynomial basis, i.e., $\operatorname{deg}(\psi _{j})=j$ for all *j*. For instance, one may take monomials $\psi _{j}(z)=z^{j}$, or Chebyshev polynomials $\psi _{j}(z)=T_{j}(z)$, suitably shifted and rescaled to account for the effective frequency range *Z*. We aim at building a rational surrogate for the state *x*, of the form

3 $$\begin{aligned} \widetilde{x}(z)= \frac{\sum_{j=0}^{S-1}p_{j}\psi _{j}(z)}{\sum_{j=0}^{S-1}q_{j}\psi _{j}(z)}. \end{aligned}$$

The coefficients $\{q_{j}\}_{j=0}^{S-1}\subset {\mathbb{C}}$ of the denominator are found as follows:

1. Build the generalized Vandermonde matrix $V\in {\mathbb{C}}^{S\times S}$, with $(V)_{ij}=\psi _{j}(z_{i})$, and compute its inverse $V^{-1}$.
2. Build the diagonal weight matrix $D\in {\mathbb{C}}^{S\times S}$, with $(D)_{ij}= (V^{-1} )_{Si}\delta _{ij}$, where $(V^{-1} )_{Si}$ is the *i*-th entry of the last row of $V^{-1}$ and $\delta _{ij}$ is the Kronecker delta: $\delta _{ij}=1$ if $i=j$ and $\delta _{ij}=0$ otherwise.
3. Compute the samples $x(z_{1}),\ldots,x(z_{S})$ and assemble the tensor $X\in {\mathbb{C}}^{n_{S}\times n_{I}\times S}$, with $(X)_{ijk}= (x(z_{k}) )_{ij}$.
4. Find the QR decomposition
  $$\begin{aligned} (X)_{ijk}=\sum_{l=1}^{S}(Q)_{ijl}(R)_{lk},\quad \text{with }Q\in { \mathbb{C}}^{n_{S}\times n_{I}\times S}\text{ and }R\in { \mathbb{C}}^{S \times S}, \end{aligned}$$
  where *Q* has orthonormal slices, i.e., $\sum_{i=1}^{n_{S}}\sum_{j=1}^{n_{I}}(\overline{Q})_{ijk}(Q)_{ijl}= \delta _{kl}$ (with a bar over a quantity, we denote the complex conjugate of such quantity). Note that *Q* and *R* can be efficiently found by computing the QR decomposition of an $(n_{S}n_{I})\times S$ *matricization* of *X*.
5. Find the vector of denominator coefficients $(q_{1},\ldots,q_{S})^{\top }\in {\mathbb{C}}^{S}$ as a (normalized) minimal eigenvector of the positive semidefinite Gramian matrix $(\overline{RDV})^{\top }RDV$. By “minimal eigenvector” we mean any eigenvector corresponding to the smallest eigenvalue. This can be done by singular value decomposition (SVD) of $RDV$, with $(q_{1},\ldots,q_{S})^{\top }\in {\mathbb{C}}^{S}$ being a minimal right singular vector of $RDV$.

Once the denominator has been found, we build the numerator coefficients $\{p_{j}\}_{j=0}^{S-1}\subset {\mathbb{C}}^{n_{S}\times n_{I}}$ by enforcing interpolation conditions. More specifically, we set

4 $$\begin{aligned} (p_{k})_{ij}=\sum _{l=1}^{S}\sum_{m=0}^{S-1}(X)_{ijl}q_{m} \psi _{m}(z_{l}) \bigl(V^{-1}\bigr)_{kl}. \end{aligned}$$

One can verify that this yields an interpolatory surrogate: $\widetilde{x}(z_{j})=x(z_{j})$ for $j=1,\ldots,S$. Also, note that $\widetilde{x}(z)$ belongs to the span of the samples $x(z_{1}),\ldots,x(z_{S})$ for all *z*, since it is a linear combination of slices of the snapshot tensor *X*.

### Adaptive frequency sampling

From the previous section, we know how to build an MRI given a set of sample points. Two more ingredients are necessary to obtain a fully fledged MOR approach with (greedy) adaptive frequency sampling: we must be able to tell if a given surrogate is “good enough” (i.e., if it satisfies the user-prescribed tolerance *ε*) and to choose where to add a new sample to improve the current surrogate (whenever the surrogate is not good enough). Several strategies for these steps are proposed by the authors in [7]. Here, we only recall one.

We start from the second point, which we reformulate as: what not-yet-sampled frequency should we add to the sampling set so as to improve the current surrogate as much as possible? Our answer is: we elect to place the next sample point at the location in *Z* where the surrogate residual is largest, namely, at

5 $$\begin{aligned} z^{\star }=\operatorname{arg}\max_{z\in Z} \bigl\Vert A(z)\widetilde{x}(z)-B(z) \bigr\Vert _{F}, \end{aligned}$$

where we use $\Vert \cdot \Vert _{F}$ to denote the $(n_{S}\times n_{I})$-Frobenius norm. In [5], it was shown that, if *A* and *B* are both polynomials of degree at most 1 in *z*, cf. (2), then (5) is exactly equivalent to the maximization of the *scalar* and *fully explicit* quantity

6 $$\begin{aligned} z^{\star }=\operatorname{arg}\max_{z\in Z} \frac{\prod_{j=1}^{S} \vert z-z_{j} \vert }{ \vert \sum_{j=0}^{S-1}q_{j}\psi _{j}(z) \vert }, \end{aligned}$$

with $\{z_{j}\}_{j=1}^{S}$ the current sampled frequencies and $(q_{j})_{j=0}^{S-1}$ the denominator coefficients of the current surrogate. Since the quantity maximized in (6) is, in general, neither convex nor bounded over *Z*, instead of finding the maximum over *Z*, we compute the maximum over just a discrete subset $Z_{\mathrm{test}}\subset Z$, with a finite (but, generally, very large) number of points.

Now we move to the first point, which we formalize as: does the current surrogate satisfy the tolerance *ε*? To begin with, we must decide on what to enforce the tolerance. One of the most practical choices is the worst-case relative approximation error

7 $$\begin{aligned} e(Z_{\mathrm{test}})=\max_{z\in Z_{\mathrm{test}}} \frac{ \Vert \widetilde{x}(z)-x(z) \Vert _{F}}{ \Vert x(z) \Vert _{F}} \leq \varepsilon. \end{aligned}$$

Note that, once again, we are seeking the maximum only over a subset of *Z* due to the unboundedness of the error on the whole *Z*, which follows from the rational nature of *x* (and of *x̃* too). A direct evaluation of $e(Z_{\mathrm{test}})$ requires computing *x* at all points of $Z_{\mathrm{test}}$, which is out of the question even if $Z_{\mathrm{test}}$ is only modestly sized. Instead, we use the following idea: under the assumption that error and residual behave somewhat similarly with respect to *z*, we can expect a large error to correspond to a large residual. As such, we replace condition (7) with the weaker version

8 $$\begin{aligned} e\bigl(\bigl\{ z^{\star }\bigr\} \bigr)= \frac{ \Vert \widetilde{x}(z^{\star })-x(z^{\star }) \Vert _{F}}{ \Vert x(z^{\star }) \Vert _{F}} \leq \varepsilon, \end{aligned}$$

where $z^{\star }$ is the point of $Z_{\mathrm{test}}$ with largest residual, see (5). This heuristic idea is much cheaper to apply, since it only requires one extra sample at $z^{\star }$. Notably, such extra sampled frequency is exactly the one that gets added to the sampling set *if* the tolerance is not attained. For this reason, the snapshot $x(z^{\star })$ does not go to waste.

### Numerical stability

When applying MRI (with or without adaptive frequency sampling), numerical instabilities may appear in two steps:^1^ when inverting the Vandermonde matrix *V* and when computing the minimal right singular vector of $RDV$.

To counteract the former issue, one could force the polynomial basis to behave “nicely” over the sampled frequencies. For instance, if $\{z_{j}\}_{j=1}^{S}$ are the Chebyshev nodes over *Z*, then the (shifted and scaled) Chebyshev polynomials yield a perfectly conditioned Vandermonde matrix. However, this is not always possible, especially considering that a greedily selected sampling set of frequencies will, in general, not be nicely placed over *Z*. This being said, by removing the (admittedly, unnecessary) constraint that the basis $\{\psi _{j}\}_{j=0}^{S-1}$ be hierarchical,^2^ we can weaken the ill-conditioning by employing the partial fraction (sometimes also called “barycentric”) basis

9 $$\begin{aligned} \psi _{0}(z)=1\quad\text{and}\quad\psi _{j}(z)= \frac{\zeta _{j}}{z-\zeta _{j}} \end{aligned}$$

($\{\zeta _{j}\}_{j=1}^{S-1}\subset {\mathbb{C}}$ being arbitrary distinct points), whose Vandermonde matrix has been empirically observed to usually have reasonable condition number. Note that (9) is a polynomial basis only after multiplication by the nodal polynomial $\prod_{j=1}^{S-1}(z-\zeta _{j})$.

Dealing with the second issue is considerably trickier, since the conditioning of the SVD of $RDV$ depends on many more factors, including, notably, the problem (1) itself. In the SVD step, the ill-conditioning manifests itself in the form of noisy minimal singular values and singular vectors, which are exactly the quantities in which we are interested. In effect, this means that the coefficients of the surrogate denominator cannot be identified robustly, leading to the appearance of so-called *spurious poles*, i.e., poles of the surrogate that do not approximate any pole of the original system. We note that similar observations have been made in [3] in the scope of scalar rational approximation. In [3], it is suggested not to trust any surrogate built from an SVD where more than one singular value is smaller than $10^{-14}\sigma _{\mathrm{max}}$, with $\sigma _{\mathrm{max}}$ being the largest singular value in question.

In summary, we can try to improve the Vandermonde-related ill-conditioning by choosing a good polynomial basis, but we *cannot* always improve the SVD-related conditioning. This justifies our approach based on partitioning the frequency range.

It is important to note that both instabilities described above can be identified on-the-fly, since they only involve fully computable quantities. Indeed, by SVD, we can directly find both the condition number of *V* and the singular values of $RDV$. Then, we can raise an “instability flag” (and interrupt the MRI algorithm) if the condition number of *V* is larger than, say, 10^14^ or if more than one singular value of $RDV$ is smaller than, say, $10^{-14}\sigma _{\mathrm{max}}$, with $\sigma _{\mathrm{max}}$ being the largest singular value of $RDV$.

Before proceeding, we remark that the issues that we have described are somewhat specific to MRI, as opposed to other MOR approaches. Indeed, in RBM, the surrogate is built (intrusively and) implicitly, so that there is no need for inversion of Vandermonde matrices nor for the identification of minimal eigenvectors. On the other hand, VF casts the rational interpolation problem in least-squares form, with well-conditioned rectangular (tall and skinny) Vandermonde matrices, but pays the price of a large oversampling.

## Automatic partitioning of the frequency range

For simplicity, we restrict our focus to the extremely common case of the frequency range $Z\subset {\mathbb{C}}$ being an interval. Generalizations to more complicated sets *Z* are not too difficult, using, e.g., 2D sparse grids on a bounding box of *Z*, cf. [4].

Assume that we are interested in building a surrogate over *Z*, with tolerance *ε* on the relative approximation error over $Z_{\mathrm{test}}\subset Z$. As such, we start the greedy-MRI approach, as described in the previous section. However, let us imagine that, at some point, the MRI algorithm raises one of the above-mentioned instability flags. Then, we partition *Z* into two parts, and try to build an *independent* surrogate over each sub-interval. If any such sub-surrogate encounters an instability, we partition further, and so on. Note that the previously computed samples of *x* should not be thrown away, but reused for the construction of the surrogate over the sub-interval to which they belong.

Since the original problem (1) is finite-dimensional, if the samples of *x* are noise-free (or if the noise is small enough), this procedure necessarily terminates, and we are left with a collection of patches that cover *Z*, each with a corresponding surrogate. Now, if we need to evaluate the overall surrogate at a new point $z\in {\mathbb{C}}$, we just find the patch to which *z* is closest (or to which *z* belongs if $z\in Z$) and evaluate the corresponding local surrogate.

The only remaining ingredient that we must specify is how to partition the frequency range. Specifically, assume that we want to split $Z=[z_{L},z_{R}]$ into two parts. Several options are possible:

- We split at the center, at $\frac{z_{L}+z_{R}}{2}$. In practice, note that, when the sampled frequencies vary over several orders of magnitude, it might make sense to take the geometric mean $\sqrt{z_{L}z_{R}}$ rather than the arithmetic one, since it corresponds to the “arithmetic center in log-scale”.
- Based on the *S* current sample points in *Z*, we split at the sample point that is closest to the mean frequency in *Z* (in the arithmetic or geometric sense). Note that, by proceeding this way, the sample at the splitting point will be shared between the two surrogates on the two sub-intervals. This, by the interpolation property, guarantees continuity of the overall surrogate across the patches, wherever a common sample point is present.

We summarize the overall procedure in Algorithm 1, where, for simplicity, we use the former “geometric mean” approach for the partitioning.

**Algorithm 1:**
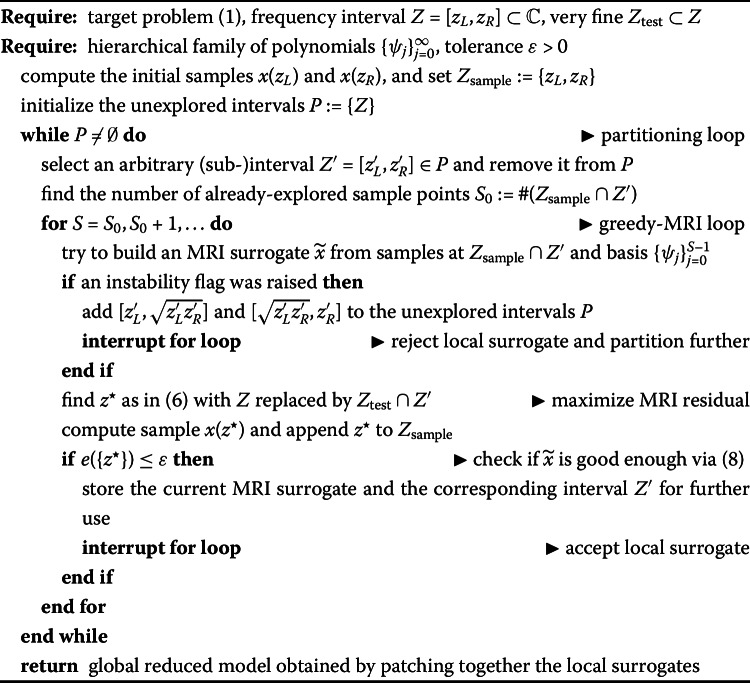
Greedy-MRI with automatic partitioning of the frequency range

To conclude the description of our proposed method, we wish to touch upon the matter of the test set $Z_{\mathrm{test}}$. In Algorithm 1, we simply choose to partition it along with the frequency range, so that each sub-interval inherits only the portion of the test set that belongs to it. However, if a local frequency interval $Z'$ becomes very small, then the corresponding test set $Z_{\mathrm{test}}\cap Z'$ might be rather tiny, or even empty, resulting in an overly early termination of the local greedy-MRI procedure. To overcome this issue, one should either start with a *very* fine test set $Z_{\mathrm{test}}$ or enrich the local test set with more elements. In our numerical experiments, we do both: we start from a fine global test set, but we also enrich the local test set with 10 logarithmically spaced points over $Z'$ whenever $Z_{\mathrm{test}}\cap Z'$ has less than 15 elements.

### Remark 1

It is rather interesting to note that our approach applies refinements both to the frequency range and to the degree of approximation (i.e., to the degrees of numerator and denominator of the rational approximant (3)). In this sense, we can draw a parallel with *hp*-adaptive strategies in finite element approximation [1], where both the spatial numerical mesh and the degree of polynomial approximation are locally adapted. However, in *hp*-adaptive finite elements, the adaptivity is usually driven by a cost-benefit criterion, whereas, here, our main concern is numerical stability.

## Numerical experiments

In this section, we showcase our MOR approach by applying it to two different systems of the form (1). Both our examples can be found among the benchmarks freely available as part of the SLICOT library. Links to the datasets and to the code used are provided at the end of the paper. Our simulations were run using Python 3.8.6 on a desktop computer with an 8-thread 3.60 GHz Intel^®^ Core^™^ i7-7700 processor.

### Impedance parameters of a transmission line

In our first example, we consider the approximation of the impedance parameters of a 2-port transmission line. After spatial discretization of the distributed circuit, we obtain a system of the form (1) with $n_{S}=256$, $n_{I}=n_{O}=2$, $A(z)=A_{0}+zE$, *B* and *C* frequency-independent matrices, and $D=0$. We consider an imaginary frequency range $Z=[10^{7}\text{i},10^{15}\text{i}]$. We seek a surrogate via MRI, using the hierarchical basis of Legendre polynomials, setting $\varepsilon =0.5\%$ and $Z_{\mathrm{test}}\subset Z$ as 10^4^ logarithmically spaced points. As described in Sect. 2, we build first a surrogate *x̃* for the state $x\in {\mathbb{C}}^{256\times 2}$ and only afterwards the surrogate $\widetilde{y}(z)=C\widetilde{x}(z)\in {\mathbb{C}}^{2\times 2}$ for the output.

The greedy-MRI method with these parameters is unable to terminate robustly, due to an instability in the SVD of $RDV$. Accordingly, we split the frequency range using Algorithm 1 at the midpoint (logarithmically speaking) $z^{\star }=10^{11}\text{i}$ and proceed recursively from there.

**Figure 1:**
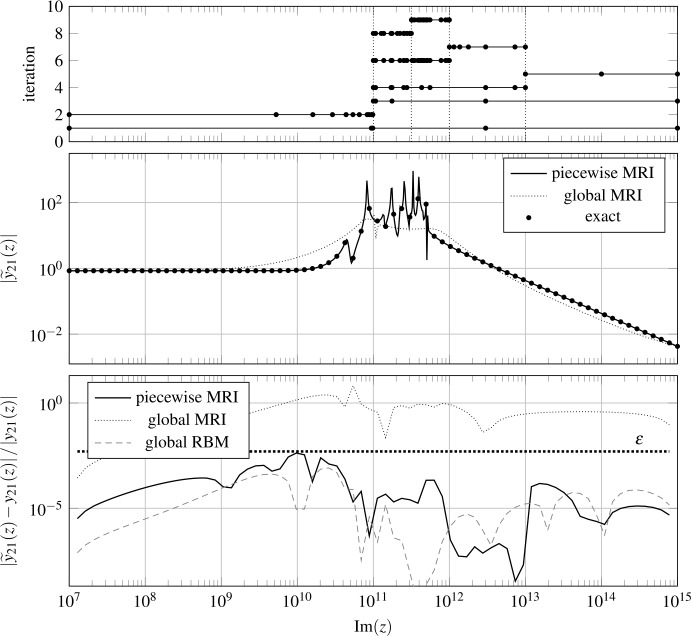
Results for the transmission line example. In the top plot, we show the sampled frequencies considered at each iteration. We denote partition boundaries by dotted vertical lines. In the middle plot, the surrogate output magnitude $\vert \widetilde{y}_{21}(z) \vert $. In the bottom plot, the relative approximation error

We show the results of the numerical experiment in Fig. 1. In Fig. 1 (top), we see what sample points are used at each iteration of Algorithm 1. We see that samples are passed on from one iteration to the next ones, and that the refinements are performed in the “interesting” frequency region around $z=10^{11}\text{i}$. In Fig. 1 (middle), we show the magnitude of the surrogate output $\vert \widetilde{y}_{21}(z) \vert $, along with some validation points $\vert y_{21}(z) \vert $, obtained by solving the original problem. We also show the relative approximation error $\vert \widetilde{y}_{21}(z)-y_{21}(z) \vert / \vert y_{21}(z) \vert $ in Fig. 1 (bottom). We see a good agreement between the overall surrogate and the exact output.

In the two latter plots, we also display the results obtained with the reduced model obtained at the end of the very first iteration of Algorithm 1, i.e., by simply keeping the MRI surrogate that is available when the algorithm is first halted for stability reasons. We see that the approximation quality is quite poor.

Moreover, in Fig. 1 (bottom), we display the results obtained with a benchmark *intrusive* MOR approach, namely, RBM, which is trained by using a relative residual estimator with the same tolerance *ε*. We do not include the RBM surrogate in Fig. 1 (middle) because it is indistinguishable from the piecewise-rational one. In the error plot, we see that the results obtained with the two methods are quite similar. However, there are some notable differences:

- The piecewise-rational approach uses 52 samples of *x* while RBM only requires 42. The main reason for this is that each new RBM sample improves the approximation quality over the whole frequency range, whereas, in Algorithm 1, each sample only affects the local interval to which it belongs. Moreover, we should note that another (minor) reason for this discrepancy is that we are enforcing the tolerance *ε* on different relative quantities: error in MRI and residual in RBM.
- Despite the higher number of samples, Algorithm 1 is approximately 55 times faster than training the RBM surrogate. This is thanks to the greedy estimator (6), which is extremely efficient when compared to the standard RBM residual estimator. A more in-depth discussion on this can be found in [7].
- Evaluating the piecewise-rational surrogate at a new frequency only involves small local reduced models, and is quite efficient. This allows for a significant time save with respect to the solution of the original problem. On the other hand, evaluating the RBM surrogate requires solving a linear system of modest size. In practice, evaluating the piecewise-rational model is about 20 times faster than solving the RBM reduced system and extracting the RBM surrogate output from it.

Before proceeding, we note that we have looked at the approximation of just a single entry of the $2\times 2$ output matrix *y*. The results for the other entries are similar.

### Structural analysis of an international space station module

As a second example, we consider the structural analysis of the Russian service module of the International Space Station. The spatial discretization of the frequency-domain linear elasticity PDE yields a system^3^ of the form (1), with $n_{S}=270$, $n_{I}=n_{O}=3$, $A(z)=A_{0}+zE$, *B* and *C* frequency-independent matrices, and $D=0$. We consider an imaginary frequency range $Z=[10^{-2}\text{i},10^{3}\text{i}]$. As in the previous example, we seek a surrogate via MRI, using the hierarchical basis of Legendre polynomials, setting $\varepsilon =0.5\%$ and $Z_{\mathrm{test}}\subset Z$ as 10^4^ logarithmically spaced points.

**Figure 2:**
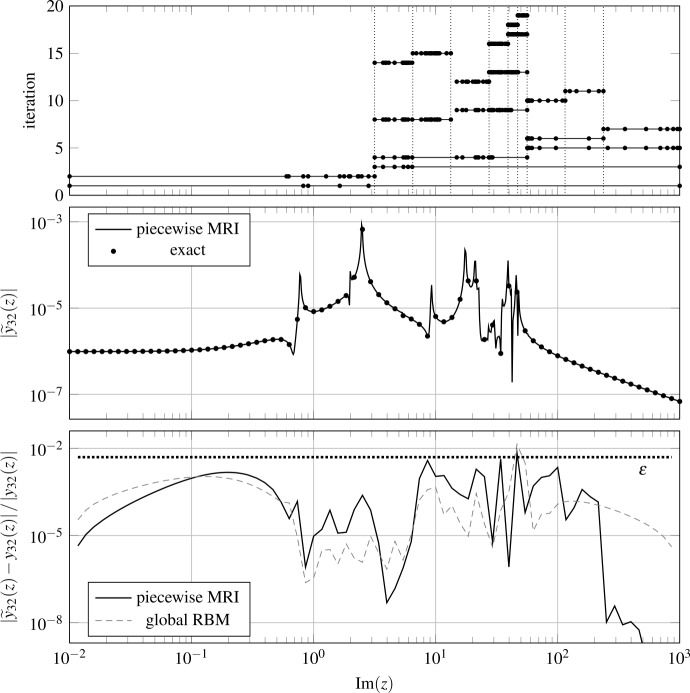
Results for the structural analysis example with (geometric) central splitting. In the top plot, we show the sampled frequencies considered at each iteration. We denote partition boundaries by dotted vertical lines. In the middle plot, the surrogate output magnitude $\vert \widetilde{y}_{32}(z) \vert $. In the bottom plot, the relative approximation error

Algorithm 1 is able to provide a piecewise-rational surrogate satisfying the tolerance, by partitioning *Z* into 10 automatically generated sub-intervals. We refer to Fig. 2 (top) for a depiction of the partitioning process. We can observe that most of the partitions are concentrated around the “busiest” frequency region $[20\text{i},60\text{i}]$.

We show in Fig. 2 (middle) the reduced model for one entry of the output, namely, $y_{32}$ (which is the one with the “most jagged” behavior). The accuracy seems good, as we also verify by looking at the relative error plot in Fig. 2 (bottom). As in the previous section, we also show the relative approximation error obtained with RBM, where, again, the training of the surrogate is done by using a relative residual estimator with the same tolerance *ε*. Once more, we see that the results of the two methods are similar. In this example, the difference in the total number of snapshots is slightly larger, with 143 samples for the piecewise-rational surrogate versus 93 for the RBM one. This being said, speedups similar to those from the previous section (∼55 and ∼20, respectively) can be observed between the training times and the evaluation times of the two approaches.

To conclude, we believe it interesting to note that the relative errors of both MRI and RBM exceed slightly the threshold *ε* at a few frequencies around $z=50\text{i}$. This is reasonable, since, after all, we are plotting the output error, whereas the tolerance is imposed on the relative state error (for MRI) or residual (for RBM), which are not necessarily upper bounds for the relative error in the output.

## Conclusions

We have presented a strategy for overcoming some instability issues that sometimes occur when applying the MRI approach to frequency response problems. Notably, we propose to replace an unstable global surrogate with a union of stable local surrogates. The partitioning of the frequency range into sub-intervals is performed automatically and adaptively. Good approximation properties have been observed in two numerical examples with rather wide frequency ranges.

The partitioning process makes the local surrogates depend on disjoint sample sets (except when samples are taken at the partition points). We observed numerically that this may increase the number of samples necessary to attain the prescribed accuracy. As a way to weaken this issue, we envision the possibility of sharing (few) samples between adjacent sub-intervals.

## Data Availability

The dynamical system matrices ($A_{0}$, *E*, *B*, and *C*) used in our numerical experiments are publicly available as part of the SLICOT library (© 2002–2021 NICONET e.V.) at http://slicot.org/20-site/126-benchmark-examples-for-model-reduction. Moreover, we have made the code used to obtain our numerical results freely available at [6].

## Footnotes

1 Note that, technically, the evaluation of the surrogate itself might also be unstable if the chosen basis $\{\psi _{j}\}_{j=0}^{S-1}$ is unstable, e.g., if it is the Lagrangian basis over uniformly spaced sample points. This issue is closely related (in fact, in some sense, equivalent) to the Vandermonde ill-conditioning that we describe.

2 If the basis is not hierarchical, the scaling matrix *D* cannot be built from the last row of $V^{-1}$ as described in Sect. 2. We refer to, e.g., [7] for the definition of *D* in the general case.

3 Note that, in the time domain, the linear elasticity PDE is of hyperbolic type, and a second-order time derivative appears in it. As such, augmentation must be applied to obtain a first-order frequency-domain system: the state *x* contains both point-displacements and point-momenta of the elastic structure.

## Abbreviations

MOR: Model Order Reduction
MRI: Minimal Rational Interpolation
PDE: Partial Differential Equation
QoI: Quantity of Interest
RBM: Reduced Basis Method
SVD: Singular Value Decomposition
VF: Vector Fitting
